# Immunopathological Dysregulation in Acute Myeloid Leukemia: The Impact of T-bet, RORγt, and FOXP3 on Disease Dynamics

**DOI:** 10.3390/cells14070528

**Published:** 2025-04-01

**Authors:** Amira M. Mohamed Mohy El-Din, Buthayna Ahmad AlShaarawy, Eman Zaghloul Kandeel, Dalia Mahmoud AlDewi, Lobna Abdel Azeem Refaat, Borros Arneth, Hussein Sabit

**Affiliations:** 1 Clinical and Chemical Pathology Department, Faculty of Medicine, Misr University for Science and Technology, Giza P.O. Box 77, Egypt; amira.mohy@must.edu.eg; 2 Clinical and Chemical Pathology Department, Girls Faculty of Medicine, Al-Azhar University, Cairo 11651, Egyptdaliaeldewi16@gmail.com (D.M.A.); 3 Clinical and Chemical Pathology Department, National Cancer Institute, Cairo University, Giza 12613, Egypt; 4 Institute of Laboratory Medicine and Pathobiochemistry, Molecular Diagnostics, Hospital of the Universities of Giessen and Marburg (UKGM), Philipps University Marburg, Baldingerstr 1, 35043 Marburg, Germany; 5 Institute of Laboratory Medicine and Pathobiochemistry, Molecular Diagnostics, Hospital of the Universities of Giessen and Marburg (UKGM), Justus Liebig University Giessen, Feulgenstr. 12, 35392 Giessen, Germany; 6 Department of Medical Biotechnology, College of Biotechnology, Misr University for Science and Technology, Giza P.O. Box 77, Egypt

**Keywords:** patterns, T helper, transcription factors, flowcytometry, acute myeloid leukemia

## Abstract

The etiology of acute myeloid leukemia (AML) is complex, including genetic and environmental abnormalities. The immune system anomalies play an essential role in the process of leukemogenesis. However, the immunopathological factors, including abnormal T helper (Th) subsets, contributing to the initiation and progression of this neoplasm, require further investigation. Considering the previously mentioned data, we decided to study the expression pattern of transcription factors T-bet, Foxp3, and ROR**γ**t that regulate Th1, Treg, and Th17, respectively, in acute myeloid leukemia with correlation to clinical and other investigation data and treatment outcomes. This study was conducted on 80 newly diagnosed patients with AML recruited from the National Cancer Institute, Cairo University, and 25 healthy control subjects. The AML patient cohort consisted of 30 females (37.5%) and 50 males (62.5%), ranging from 18 to 74 years old. The control group was 8 females (32%) and 17 males (68%), with ages ranging from 23 to 40 years old. Samples were provided from the bone marrow of donor cases for allogeneic bone marrow transplantation. The diagnosis of acute myeloid leukemia was based on morphologic and cytochemical evaluation, immunophenotyping, and complementary cytogenetics according to WHO criteria. Upshift from the normal T-bet intensity of power (MFI), ROR**γ**t^+^ CD4^+^ T lymphocyte frequency (%) with downshift from the normal FOXP3 intensity of power (MFI), may suggest a state of inflammation. In contrast, an upshift from the normal FOXP3^+^ CD4^+^ T lymphocyte frequency (%) may reflect a state of immunosuppression in the bone marrow microenvironment of AML. Combined, they constitute a sophisticated scenario of immunological disorder in AML. Co-expression of T-bet and ROR**γ**t transcription factors in CD4^+^ T lymphocytes in both normal and AML groups may suggest CD4^+^ T lymphocyte plasticity.

## 1. Introduction

Acute myeloid leukemia (AML) is the most common acute leukemia affecting adults, and its incidence increases with age. Globally, leukemia accounts for approximately 3.5% of all cancer cases. Among these, AML is the most common subtype in adults [1]. However, precise global statistics on AML’s proportion relative to all cancers are limited. In the United States, AML represents about 1% of all cancer diagnoses. Similarly, in the United Kingdom, AML accounts for less than 1% of all new cancer cases [2]. Over the last three decades, the prognosis of AML has improved only modestly, with 5-year overall survival rates rising from ~15–20% in the 1990s to ~30% today for adults under 60, largely due to advances in supportive care, optimized chemotherapy, and allogeneic stem cell transplantation. While modern diagnostics (e.g., next-generation sequences (NGS) and minimal residual disease (MRD) monitoring) enable precision risk stratification and guide therapies, these tools have not overcome the biologic aggressiveness of many AML subtypes or the lack of effective treatments for resistant disease, resulting in minimal population-level survival improvements [3]. Thus, despite incremental progress in younger, fitter patients, AML prognosis remains poor overall, underscoring decades of therapeutic stagnation for most patients. Studies focused on the immunological background of bone marrow that provoked leukemic clones, drug resistance, and subsequent relapses [4]. Understanding the different CD4^+^ T cells subsets may reinstate the immunological cure of AML. The mechanisms adopted by leukemic clones that drive the immune response have not yet been underexplored [5].

Chronic proinflammation plays a significant role in the pathogenesis and progression of AML by shaping a dysregulated bone marrow microenvironment that supports leukemic cell survival, proliferation, and therapy resistance. In AML, inflammatory cytokines such as IL-1β, IL-6, TNF-α, and TGF-β are often elevated, activating oncogenic signaling pathways (e.g., NF-κB and STAT3) that drive leukemogenesis, suppress apoptosis, and promote chemoresistance [6].

Immune system disorder has been shown in the pathogenesis of AML. T cells are immune, essential for anti-tumor immunity, and eliminate AML cells through releasing cytokines and cytotoxic substances. Parallelly, AML cells influence T cell differentiation and proliferation and play an immunosuppressive role by releasing inhibitory cytokines or other mechanisms. The immunosuppressive tumor microenvironment (TME) in cancers like AML and breast cancer drives immune cell dysfunction, particularly T cell exhaustion, by creating hypoxic, nutrient-deprived conditions and upregulating inhibitory signals (e.g., PD-1/PD-L1 and adenosine). A 2024 study identifying TOX, CD39, and CXCR5 as novel markers of exhausted CD8^+^ T cells in breast cancer highlights how chronic antigen exposure in the TME epigenetically silences effector genes (via TOX) and disrupts metabolic pathways, mirroring mechanisms in AML where leukemic blasts and stromal cells overexpress PD-L1 and CD39/CD73, promoting adenosine-mediated T cell suppression [7].

T helper (Th) cells are pivotal in the T cell immune system network. Previous studies on Th cells are limited to Th1 or Th2 subset [8]. Accumulating evidence indicates an imbalanced Th1/Th2 involved in the pathogenesis of solid tumors [9] as well as hematological malignancies [10]. T-bet, a T-box transcription factor, is expressed in CD4^+^ T lymphocytes committed to Th1 T-cell development and may participate in immunoglobulin class switching in B lymphocytes [11].

Th17 and Treg are considered other paired Th subsets, and Th17/Treg imbalance is found in many diseases [12]. Tregs are characterized by constitutive expression of high-level CD25, and forkhead-winged helix transcription factor (Foxp3) is essential for Tregs development and functions. Tregs maintain natural self-tolerance and control immune responses to foreign antigens [8]. Several research studies have shown elevated levels of Tregs in several hematological diseases, including AML [5].

Th17 cells have a regulatory role in normal hematopoiesis [13]. It has been established that Th17 cells participate in some autoimmune diseases and tumors [14]. Retinoic acid-related orphan nuclear receptor gamma t (ROR-γt) is a transcription factor that is considered to be essential for the initiation and maintenance of the Th17 cell lineage [15].

The present study aims to investigate the expression pattern of different transcription factors T-bet, Foxp3, and RORγt that regulate Th1, Treg, and Th17, respectively, in AML, with correlation to clinical and other investigation data and treatment outcomes.

## 2. Subject and Methods

This study involved 80 de novo AML patients recruited from the National Cancer Institute, Cairo University, and 25 age- and sex-matched healthy control subjects who were donors for allogeneic bone marrow transplantation. The AML cohort comprised 30 females (37.5%) and 50 males (62.5%) aged 18 to 74. The control group comprised 8 females (32%) and 17 males (68%), with ages ranging from 23 to 40 years.

The diagnosis of AML was confirmed using a combination of morphologic examination, cytochemical staining, immunophenotyping, cytogenetic analysis, and molecular studies in selected cases, all following the WHO criteria 2016 [16].

### 2.1. Procedures

Patients underwent a comprehensive assessment, including a detailed medical history with a focus on leukemia-associated symptoms such as fever, fatigue, bleeding tendencies, and bone pain. A thorough clinical examination was performed, emphasizing signs of leukemia involvement, including pallor, purpuric eruptions, hepatomegaly, splenomegaly, lymphadenopathy, and central nervous system manifestations. Laboratory investigations included a complete blood count (CBC), analyzed using the Sysmex XN1000 [Sysmex Corporation, Kobe, Japan] and Leishman-stained peripheral blood smears to evaluate the differential leukocyte count and the percentage of peripheral blood blast cells. Bone marrow (BM) aspiration and examination of Leishman-stained smears were conducted to assess BM blast cell percentage. Cytochemical staining for myeloperoxidase was performed on marrow smears to classify leukemia subtypes further. Immunophenotyping of blast cells was carried out using erythrocyte-lysed whole BM and a panel of monoclonal antibodies (MoAb) labeled with FITC/PE/ECD/PC5.5/PC7/APC for diagnostic purposes, including markers such as CD45, CD34, CD38, CD117, HLA-DR, CD13, CD33, CD64, CD13, CD11C, CD123, CD11B, CD61, CD41 CD10, CD10, CD22, cCD79a, CD3, CD4, CD8, CD5, CD7, CD56, and others (Agilent Technologies Dako, Glostrup, Denmark) (Beckman Coulter, Miami, FL, USA). The gating strategy involved initial exclusion of debris and dead cells using FSC versus SSC and viability dyes. AML blasts were identified based on CD45 versus SSC gating, characterized by low CD45 expression and low SSC, with further classification using CD34, CD117, HLA-DR, and aberrant markers such as CD7 and CD56. Further classification of AML subtypes was performed based on specific marker expression: AML with minimal differentiation (CD34^+^ CD117^+^ HLA-DR^+^ but lacking lineage-specific markers), AML without maturation (CD34^+^ CD117^+^ HLA-DR^+^ with myeloid markers CD13/CD33), AML with maturation (CD13+ CD33^+^ CD15^+^ CD64^+^), acute promyelocytic leukemia (APL) (CD34^-^ HLA^-^DR^-^ CD117^+^ CD33^+^ CD64^+^ with strong CD13 and CD56 expression), AML with monocytic differentiation (CD64^+^ CD14^+^ CD4^+^ CD11c^+^), and AML with myelodysplasia-related changes (heterogeneous expression of myeloid markers and frequent aberrant CD7 expression).

Lymphocytes were first gated based on their low SSC, moderate FSC, and intermediate to high CD45 expression. T cells were gated by selecting lymphocytes with low SSC and moderate FSC, followed by CD3^+^ gating to isolate total T cells. Subsets were distinguished as CD3^+^ CD4^+^ T-bet^+^ for Th1 Cells, CD3+ CD4+ RORγt+ for Th17 cells, and CD3^+^ CD4^+^ CD25^+^ FOXP3^+^ for regulatory T cells.

Markers were considered positive when ≥20% of gated blasts expressed it, except for CD34 and MPO, where ≥10% was sufficient for positivity. The markers’ mean fluorescence intensity (MFI) was determined using a six-color Navios Coulter Flow Cytometer (Beckman Coulter, Miami, FL, USA).

### 2.2. Methods

For each case, 2 mL of peripheral blood was collected into ethylene diamine tetra-acetic acid (EDTA) tubes (1.2 mg/mL) as an anticoagulant for performing the complete blood count (CBC) and preparing Leishman-stained peripheral blood (PB) smears. Bone marrow aspiration was performed under strict aseptic conditions for both patients and control subjects. A few drops of the aspirate were used to prepare smears for Leishman and myeloperoxidase (MPO) staining for patients. Another 2 mL of aspirate was collected into two sterile tubes: K-EDTA for flow cytometry and lithium heparin for cytogenetic analysis. For control subjects, a few drops of the aspirate were used for Leishman staining, and 2 mL were placed into a sterile K-EDTA tube for flow cytometry.

T-bet expression was detected using fluorescein-conjugated (FITC) monoclonal mouse IgG (Clone 525803) (R&D Systems, Minneapolis, MN, USA), prepared in 1 mL saline with 0.5% bovine serum albumin (BSA) and <0.1% sodium azide. RORγt expression was assessed using allophycocyanin-conjugated (APC) monoclonal mouse IgG2B (Clone 600380) (R&D Systems, Minneapolis, MN, USA), similarly prepared in saline with 0.5% BSA and <0.1% sodium azide. FOXP3 expression was measured with phycoerythrin-conjugated monoclonal mouse IgG (PE) (Clone 1054C) (R&D Systems, Minneapolis, MN, USA), also in saline with 0.5% BSA and <0.1% sodium azide.

Phosphate-buffered saline (PBS) (8.0 g/L NaCl, 0.2 g/L KCl, 1.15 g/L NaH_2_PO_4_, and 0.2 g/L KH_2_PO_4_, pH adjusted to 7.3 ± 0.2) was stored at 4 °C for use without contamination. Lysing solution (1.5 mmol/L NH_4_Cl, 100 mmol/L KHCO_3_, and 10 mmol/L tetra Na-EDTA, pH 7.2) was prepared in distilled water. Negative isotypic controls were included to assess background fluorescence intensity and non-specific antibody binding.

T-bet and RORγt were evaluated in the lymphocyte population, considering the percentage positivity as well as median fluorescent intensity (MFI). For surface marker analysis, the EDTA anticoagulated BM sample was diluted with PBS to adjust the cell count. For each sample, a set of tubes was labeled for all the MoAbs to be used, including one tube for the appropriate negative isotypic-matched control MoAb. 50 µL of diluted samples was delivered in each tube. 5 µL of each MoAb, as well as of the isotypic negative control MoAb, was added to the respective tubes. The tubes were vortexed and incubated in the dark at room temperature for 15 min. 1.5 mL lysing solution was added to each tube. The tubes were centrifuged at 3000 rpm for 5 min, and the supernatant was discarded. 2 mL of PBS, as a wash buffer, was added to each tube and mixed thoroughly. The tubes were centrifuged at 3000 rpm for 5 min, and the supernatant was discarded. The cells were washed once with 2 mL PBS, with centrifugation, and discarding the supernatant. Cells were suspended in 300 µL PBS to be processed by the FCM. Negative control samples were introduced in the machine. The autoflourescence region for PE stain was adjusted for each sample.

For intracellular marker analysis of FOXP3, surface staining for CD25 was first, as previously described for surface staining. Then, add 100 μL permeabilization buffer. Incubate for 10 min. Add 5 μL of intracellular monoclonal antibody. Wash the cells with 2 mL PBS, with centrifugation, and discarding the supernatant. Cells are suspended in 300 µL PBS to be processed by the FCM.

A minimum of 100,000 events were studied. Gating was done on the lymphocyte population based on forward, side scatter properties, and CD45. The excitation wavelength was 488 nm Argon laser.

### 2.3. Data Analysis

Data analysis was conducted using IBM SPSS Advanced Statistics (version 22) (SPSS Inc., Chicago, IL, USA). Depending on the distribution, numerical data were presented as either the mean and standard deviation or the median and range. For comparisons of customarily distributed numeric variables, the Student’s *t*-test was used, while for non-normally distributed numeric variables, the Mann–Whitney test was applied. Categorical variables were expressed as frequencies and percentages, and comparisons between categorical data were performed using the chi-square test or Fisher’s exact test, depending on the conditions. All statistical tests were two-tailed to ensure robustness in the results.

### 2.4. Ethical Approval

This study was approved by the Institutional Review Board of the National Cancer Institute (IRB-NCI), IRB Approval No. 2106-303-022

Participants were provided with detailed information regarding the study’s purpose, procedures, potential risks, and benefits. Consent was documented through signed forms, and patients retained the right to withdraw at any stage without any impact on their medical care.

## 3. Results

This study was conducted on 80 de novo AML patients and 25 age- and sex-matched healthy control participants. AML patients were 30 females (37.5%) and 50 males (62.5%), aged 18 to 74. The control group consisted of 8 females (32%) and 17 males (68%), whose ages ranged from 23 to 40 years old. Control samples were provided from the bone marrow of donor cases for allogeneic bone marrow transplantation. There was no statistically significant difference between the control group and AML patient group regarding age and gender distribution (Table 1).

The histological laboratory parameters were measured for both groups. There is a statistically highly significant increase in total leucocytic count (TLC) and BM blasts (*p* < 0.001). At the same time, there is a highly significant decrease in Hb and platelet count in the AML group compared with the standard control group (*p* < 0.001). Data are represented in Table 2.

The study on acute myeloid leukemia (AML) patients classified by the French-American-British (FAB) system revealed the following distribution: 26.3% had M1, 17.5% had M2, 33.8% had M4, and 22.5% had M5b.

T-bet MFI and RORγt^+^ CD4^+^ cells % were significantly higher in AML patients compared to the control group, suggesting increased immune activation. FOXP3^+^ CD4^+^ cells % also showed a marked increase in AML patients, indicating potential immune system dysregulation. However, T-bet^+^ cells %, RORγt MFI, and co-expression of RORγt and T-bet did not differ significantly, implying that these markers might not be as strongly associated with AML progression (Table 3).

**Figure 1:**
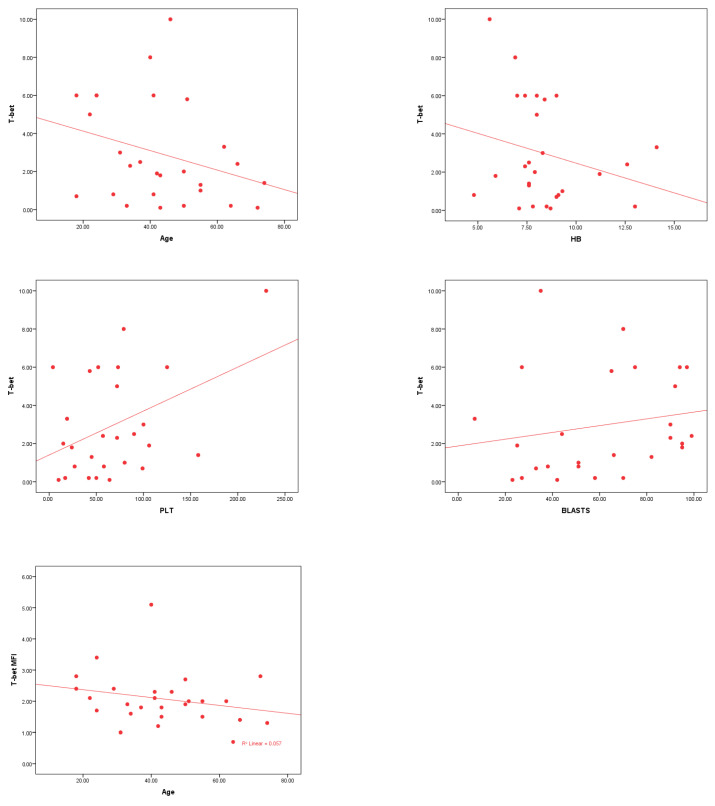
The analysis reveals significant correlations between T-bet^+^ CD4^+^ T lymphocytes % and various clinical parameters in AML patients. A highly significant negative correlation exists between T-bet^+^ CD4^+^ T lymphocytes % and age (*p* = 0.007) and a significant negative correlation with Hb levels (*p* = 0.029). A highly significant positive correlation is observed between T-bet^+^CD4^+^ T lymphocytes % and platelet count (*p* = 0.008), as well as with BM blasts % (*p* = 0.004). T-bet MFI also shows a highly significant negative correlation with age (*p* = 0.005).

T-bet^+^ cells and T-bet MFI show significant correlations with age, with negative correlations of −0.298 (*p* = 0.007) and −0.311 (*p* = 0.005), respectively. Both markers also correlate with PLT and BM blasts, with T-bet^+^CD4^+^ T cells showing a positive correlation with PLT (0.292, *p* = 0.008) and BM blasts (0.315, *p* = 0.004). In contrast, no significant correlations were found between T-bet^+^ CD4^+^ cells, T-bet MFI and TLC, Hb, or PLT MFI (Table 4 and Figure 1).

**Figure 2:**
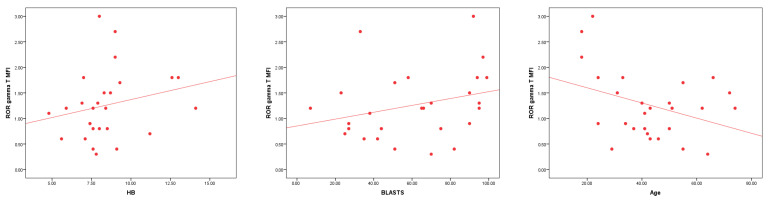
The correlations between RORγt MFI and various clinical parameters in AML patients. A highly significant positive correlation is observed between RORγt MFI and Hb levels (*p* = 0.006) and with BM blasts % (*p* = 0.001). Additionally, a significant negative correlation is found between RORγt MFI and age (*p* = 0.013), suggesting varying immune system responses in relation to these clinical factors.

RORγt^+^CD4^+^cells and RORγt MFI show varying correlations with clinical parameters in AML patients. RORγt MFI has a significant negative correlation with age (r = −0.278, *p* = 0.013) and a significant positive correlation with Hb (r = 0.307, *p* = 0.006) and BM blasts % (r = 0.349, *p* = 0.001). RORγt^+^CD4^+^cells showed weaker or non-significant correlations with age, TLC, PLT, and BM blasts % (Table 5 and Figure 2).

**Figure 3:**
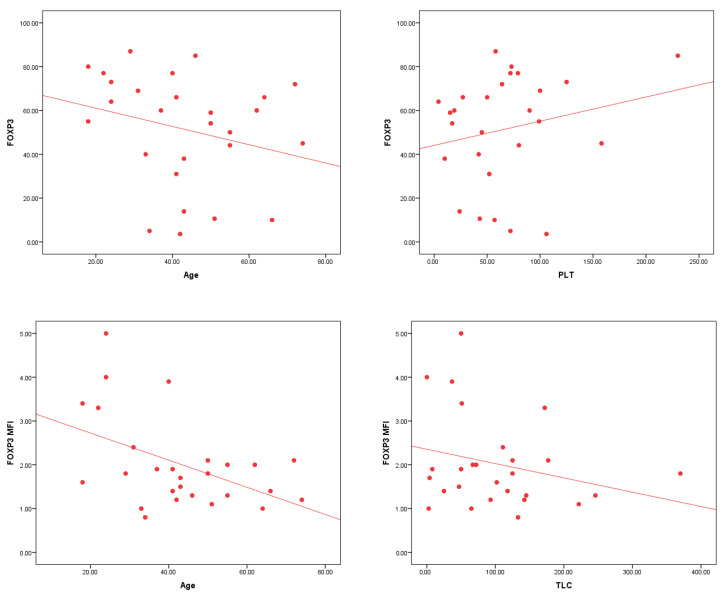
The significant correlations between FOXP3^+^ CD25^+^ CD4^+^ T lymphocytes % and FOXP3 MFI with clinical parameters in AML patients. There is a highly significant negative correlation between FOXP3^+^ CD25^+^ CD4^+^ T lymphocytes % (*p* = 0.001) and FOXP3 MFI (*p* = 0.001) with age. FOXP3^+^ CD25^+^ CD4^+^ T lymphocytes % shows a significant positive correlation with platelet count (*p* = 0.036), while FOXP3 MFI is significantly negatively correlated with TLC (*p* = 0.023).

FOXP3^+^CD25^+^CD4^+^ cells and FOXP3 MFI significantly correlate with several clinical parameters in AML patients. Both markers exhibit a significant negative correlation with age (r = −0.357, *p* = 0.001 for FOXP3^+^; r = −0.373, *p* = 0.001 for FOXP3 MFI). FOXP3 MFI also shows a significant negative correlation with TLC (r = −0.254, *p* = 0.023), while FOXP3^+^ cells are positively correlated with platelet count (r = 0.235, *p* = 0.036) (Table 6 and Figure 3).

**Figure 4:**
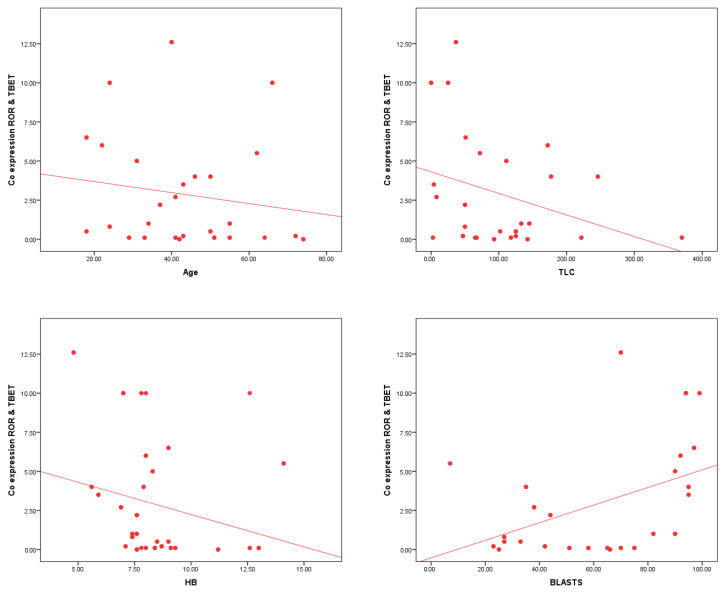
The significant correlations between T-bet^+^ RORγt^+^ CD4^+^ T lymphocytes % and clinical parameters in AML patients. A significant negative correlation is observed between T-bet^+^ RORγt^+^ CD4^+^ T lymphocytes % and age (*p* = 0.019) as well as Hb levels (*p* = 0.016), and a highly significant negative correlation with TLC (*p* = 0.008). Furthermore, a highly significant positive correlation is found between T-bet^+^ RORγt^+^ CD4^+^ T lymphocytes % and BM blasts % (*p* < 0.001).

The co-expression of RORγt and T-bet shows significant correlations with several clinical parameters in AML patients. A significant negative correlation is observed with age (r = −0.263, *p* = 0.019), TLC (r = −0.296, *p* = 0.008), and Hb levels (r = −0.268, *p* = 0.016). Additionally, a highly significant positive correlation is found with BM blasts % (r = 0.444, *p* < 0.001) (Table 7 and Figure 4).

The distribution of T-bet^+^CD4^+^ T lymphocytes and T-bet MFI across various clinical factors in AML patients is represented in Table 8. Gender differences in T-bet^+^CD4^+^ T cells and T-bet MFI are not statistically significant, with median values of 2.5 for females and 1.9 for males and mean MFI values of 2.16 and 2.03, respectively. For FAB classification, significant variation is observed in the T-bet levels for M1, M4, and M5b subtypes compared to M2, with M1 showing higher median values (2.4). The *p*-values indicate statistically significant differences for FAB subtypes (T-bet: <0.001).

**Figure 5:**
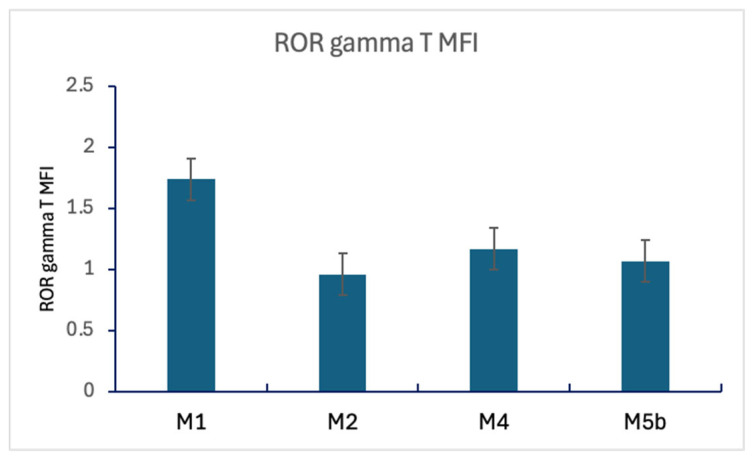
RORγt^+^ CD4^+^ T lymphocytes % and RORγt MFI in FAB studied groups: RORγt^+^ CD4^+^ T lymphocytes percentage is significantly higher in M1 AML than in other studied FAB groups (*p* = 0.018). RORγt MFI is significantly higher in M1 AML than in other FAB groups (*p* < 0.001).

RORγt^+^CD4^+^ T cells % and RORγt MFI show significant differences across various groups, particularly in FAB classification. FAB M1 displays the highest RORγt^+^CD4^+^ T cell % and MFI, with substantial variations across all categories (Table 9 and Figure 5).

FOXP3^+^CD25^+^CD4^+^ cell percentage and FOXP3 MFI values vary across genders and FAB groups, with no significant differences between males and females for either measure. The M1 FAB group shows higher FOXP3 MFI than others but no significant difference (*p* = 0.071) (Table 10).

**Figure 6:**
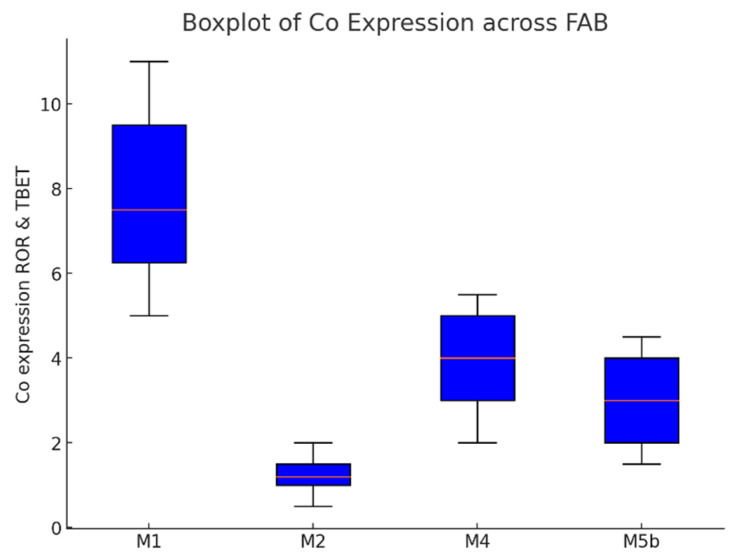
CD4^+^ T lymphocytes co-expressing T-bet and RORγt in studied FAB groups: A highly significant increase in CD4^+^ T lymphocytes co-expressing T-bet and RORγt is observed in M1 AML compared to other studied FAB groups (*p* < 0.001).

RORγt and T-bet co-expression levels vary significantly across FAB subtypes and survival rates, while gender differences are not statistically significant (*p* = 0.182). Among FAB subtypes, M1 exhibits the highest expression (6.0 median), whereas M2, M4, and M5b show significantly lower values (*p* < 0.001) (Table 11 and Figure 6).

**Figure 7:**
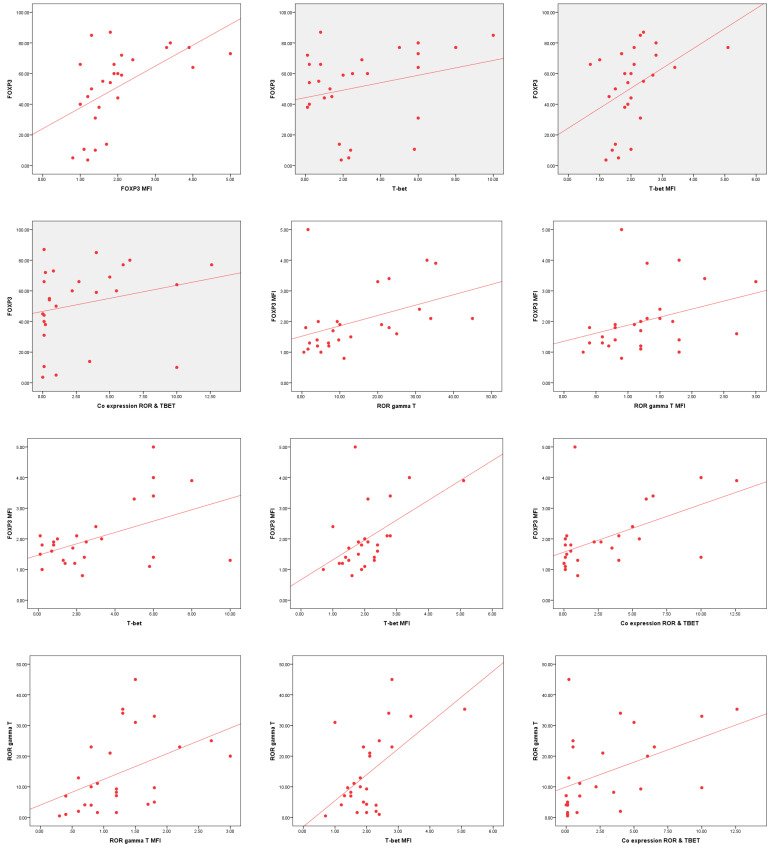

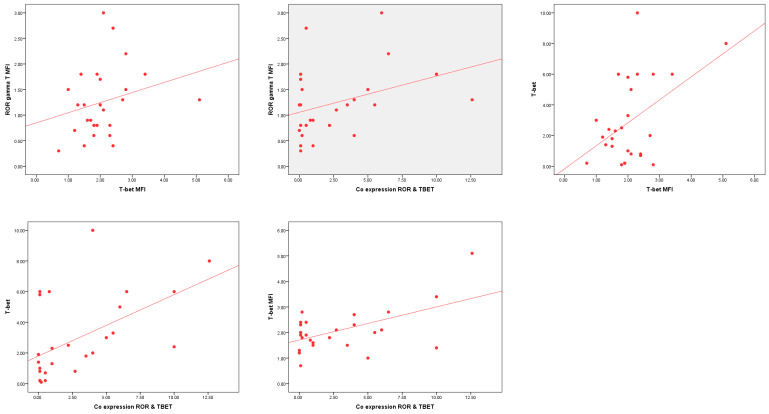
The correlation analysis in AML patients reveals highly significant positive relationships between FOXP3, RORγt, and T-bet expression levels and their median fluorescence intensity (MFI). FOXP3^+^CD25^+^CD4^+^ T lymphocytes % show strong correlations with FOXP3 MFI, T-bet^+^CD4^+^ T lymphocytes %, T-bet MFI, and RORγt^+^Tbet^+^CD4^+^ T lymphocytes % (*p* < 0.001), indicating a potential regulatory link between these markers. Similarly, FOXP3 MFI is significantly correlated with RORγt^+^CD4^+^ T lymphocytes %, RORγt MFI, T-bet^+^CD4^+^ T lymphocytes %, T-bet MFI, and RORγt^+^Tbet^+^CD4^+^ T lymphocytes % (*p* < 0.001). Additionally, RORγt^+^CD4^+^ T lymphocytes % and RORγt MFI exhibit strong correlations with T-bet MFI and RORγt^+^Tbet^+^CD4^+^ T lymphocytes %, reinforcing their interplay in immune modulation. T-bet^+^CD4^+^ T lymphocytes % and T-bet MFI are also positively correlated with RORγt^+^Tbet^+^CD4^+^ T lymphocytes %, emphasizing their combined role in AML immune response regulation (*p* < 0.001). These findings highlight the interconnected regulation of FOXP3, RORγt, and T-bet in AML patients, suggesting potential implications for disease progression and therapeutic targeting.

Significant correlations were observed among FOXP3, RORγt, and T-bet expression and their median fluorescence intensity (MFI). FOXP3 MFI showed a strong positive correlation with FOXP3 % (r = 0.642, *p* < 0.001) and T-bet MFI (r = 0.503, *p* < 0.001). RORγt % correlated significantly with RORγt MFI (r = 0.537, *p* < 0.001) and T-bet MFI (r = 0.411, *p* < 0.001), indicating potential interplay in immune regulation. Co-expression of RORγt and T-bet % was significantly associated with all measured markers, particularly FOXP3 MFI (r = 0.586, *p* < 0.001) and RORγt % (r = 0.565, *p* < 0.001). These findings suggest a complex regulatory network among FOXP3, RORγt, and T-bet in immune modulation (Table 12 and Figure 7).

**Figure 8:**
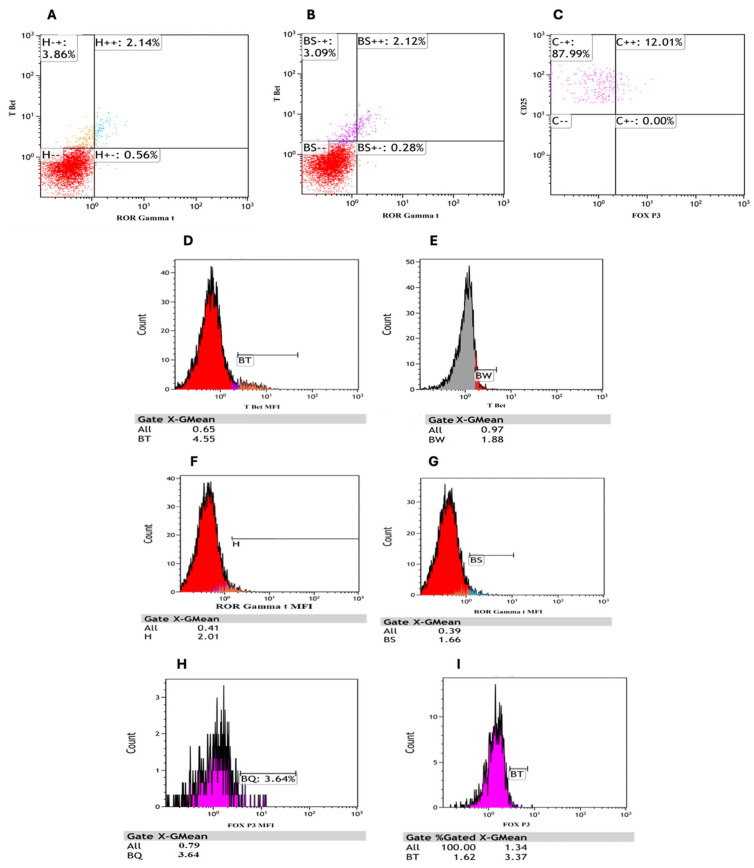
The expression of T-bet, RORγt, and FOXP3 in CD4^+^ T lymphocytes from AML patients and control subjects using dot plots and histograms. Red population gated on CD3^+^ CD4^+^ T lymphocytes while purple population gated on CD3^+^ CD4^+^ CD25^+^ T lymphocytes. (**A**) shows a dot plot of T-bet and RORγt expression in AML patients, indicating the proportion of different populations. (**B**) presents a dot plot of T-bet and RORγt expression in control subjects, demonstrating a different distribution. (**C**) displays a dot plot of CD25 and T-bet expression in AML patients. (**D**) shows the histogram of T-bet intensity in control subjects, providing a comparison. (**E**) shows the histogram of RORγt intensity in AML patients, highlighting its expression profile. (**F**) provides the histogram of RORγt intensity in control subjects for comparison. (**G**) illustrates a dot plot of FOXP3 expression in CD4^+^ CD25^+^ T lymphocytes in AML patients, highlighting regulatory T cells. (**H**) shows the histogram of FOXP3 expression intensity in AML patients’ CD4^+^ CD25^+^ T lymphocytes. (**I**) presents the histogram of FOXP3 expression in control subjects’ CD4^+^ CD25^+^ T lymphocytes, providing a baseline for comparison.

T-bet, RORγt, and FOXP3 expression profiles in CD4^+^ T lymphocytes from AML patients and control subjects were analyzed (Figure 8). Significant differences in the distribution and expression levels between AML patients and controls were observed. These results suggest immune modulation alterations in AML that could impact disease progression and therapeutic targeting.

## 4. Discussion

Acute myeloid leukemia (AML) is a serious hematological stem cell neoplasm with diverse genetic aberrations, phenotype, clinical presentation, and response to therapy [17]. The immune system anomalies play important roles in the process of leukemogenesis. However, the immunopathological factors, including the abnormal T helper (Th) subsets, leading to the initiation and progression of this serious neoplasm, need a lot of studies to be well understood [18,19].

T-bet is a master transcription factor of Th1 cells that aids the elimination of malignant cells by activating macrophages in a IFNγ dependent way [20]. Retinoic acid-related Orphan nuclear receptor gamma t (RORγt) is a transcription factor critical for initiating and maintaining the Th17 cell lineage and regulating the differentiation of the Th17 subset [21]. Regulatory T cells, a subgroup of CD4^+^ T cells, are characterized by positivity for CD4, CD25, and FOXP3 [22].

In the present study, we evaluated the expression patterns of some CD4^+^ T lymphocyte transcription factors (T-bet, RORγt, and FOXP3) that regulate Th1, Th17, and Treg in the BM of de novo AML cases. The expression patterns include the frequency of CD4^+^ T lymphocytes expressing the marker percent and intensity of marker expression (MFI) in the immune cells of the AML bone marrow microenvironment.

The present study revealed a statistically significant increase in the TLC and BM blasts % and a statistically significant decrease in platelet count and hemoglobin level in the AML group. These results are primarily due to increased blast cell infiltration in the bone marrow with dissemination in the peripheral blood and impaired hematopoiesis with displacement of normal blood cell formation by malignant blasts. Previous studies reported the presence of leukocytosis in AML patients [23], and AML should be suspected in anyone with unexplained cytopenias (decreased cell count of white blood cells, hemoglobin, or platelets), the presence of circulating blast cells in the peripheral blood, easy bruising or bleeding, as well as recurrent infections [17].

Further, we compared CD4^+^ T lymphocyte positive % and MFI of the studied markers in the bone marrow microenvironment in the AML and control group. A statistically significant increase of T-bet MFI, RORγt ^+^CD4^+^cells %, and FOXP3^+^ CD25^+^ CD4^+^ cells %, along with a statistically significant decrease of FOXP3 MFI in the AML group was observed. These results indicate that there is an upshift of RORγt ^+^CD4^+^cells % and FOXP3^+^ CD25^+^ CD4^+^ T cells, which are under the control of cell recruitment and activation mechanisms in AML. Also, there is an upshift of T-bet but a downshift of FOXP3 intensities of power (MFI) that are under the control of their specific gene expression machinery. On the other hand, there is no change in the T-bet^+^ CD4^+^ T cell frequency or RORγt power intensity in AML. The frequency of the cells expressing a marker is different and separate from the specific marker expression intensity; hence, they have other effects on the pathogenesis of AML.

We may suggest that there is an impact of these shifted frequencies of CD4^+^ T lymphocytes positive to the mentioned transcription markers and the shifted power of the marker expression in the pathogenesis of AML. The highly increased T-bet MFI, RORγt^+^ CD4^+^cells %, and the significant decrease of FOXP3 MFI suggest a state of inflammation reaction, while the highly significant increased FOXP3^+^ CD25^+^ CD4^+^ cells % may indicate an associated immunosuppression status. Both inflammations associated with immunosuppression may suggest a sophisticated deviated immunological status that may have an impact on the pathogenesis of AML.

In line with our results, [5] concluded that the immunosuppressive state that allowed the occurrence of AML is mostly the result of the increased Treg (FOXP3^+^ CD25^+^ CD4^+^) T cells’ frequency. However, they reported a decrease in regarding Th1 (IFNγ^+^ CD4^+^) and Th17 (IL17^+^ CD4^+^) T cells. This conflict may be due to different markers used in detecting these cells in their study other than the markers used in detecting these cells in our research (Tbet^+^ CD4^+^ for Th1 and RORγt^+^ CD4^+^ for Th17).

Meanwhile, Wang, Hu [24] mentioned that the percentage of circulating Th1 cells was significantly decreased in newly diagnosed AML patients compared with controls; Th17 cells were significantly increased in newly diagnosed patients compared with controls.

Our results regarding the percentage of RORγt^+^ CD4^+^ T cells representing Th17 align with Abousamra, Salah El-Din [25] and Han, Ye [26]. Both studies stated the increase in the percentage of Th17 in AML. However, they disagreed on whether Th17 in AML was a good or bad prognostic parameter, where [25] stated that Th17 increases overall survival. At the same time, [26] considered the increase of Th17 percent to be a poor prognostic factor. Th17 cells, among other cell types, are increased in AML patients, as stated by [27].

Our data indicated a significant decrease in the T-bet^+^ CD4^+^ T lymphocyte frequency and T-bet intensity of expression, and this may highlight the relatively deficient immune response with an increase in age. The steady decline in the production of fresh, naïve T cells, more restricted T cell receptor (TCR) repertoire, and weak activation of T cells are some of the effects of aging [28].

The highly significant positive correlation of T-bet^+^ CD4^+^ T lymphocytes % with platelet count may indicate an impact of these cells on thrombopoiesis and, hence, blood platelets and/or an impact of platelets on these cells. The platelets’ concentration dependently enhanced IFNγ production by CD4^+^ T cells but attenuated their proliferation. Platelets enhanced the production of IL-10 and cytokines characteristic for type 1 T helper (Th1) (IFNγ/TNFα) and Th17 (IL-17) cells [29].

The correlation between RORγt^+^ CD4^+^ T cells % and RORγt MFI with age and hematological parameters was assessed. Although RORγt^+^ CD4^+^ T cells % showed a non-significant negative correlation with age, RORγt MFI showed a highly significant negative correlation with the increase of age. It was previously reported [30] that there is a significant decrease in the frequency of Th17 cells in the elderly. They explained a possible mechanism for this decrease is reduced expression of the transcription factor RORγt that is essentially involved in promoting IL-17 production. Furthermore, IL-17 significantly alters erythropoiesis by modifying erythroid progenitor cell frequencies, stimulating BFU-E, and suppressing CFU-E in the bone marrow and spleen. This led to increased peripheral blood CFU-E and reticulocytosis, indicating effective erythropoiesis and mobilization of progenitors [31].

Our results demonstrate a negative correlation between Treg (FOXP3^+^ CD25^+^ CD4^+^ T cells) frequency and FOXP3 MFI with age and a positive impact of Treg frequency on thrombopoiesis. At the same time, FOXP3 intensity negatively affects leucopoiesis, potentially contributing to deficient immunity and recurrent infections in AML patients. These findings align with increasing evidence showing Treg dysfunction in aged patients, suggesting that immune therapies targeting Tregs could benefit diseases like cancer and autoimmune disorders [32]. Moreover, Foxp3 is essential for proper megakaryopoiesis and platelet function, including platelet spreading and release [33], further highlighting the role of Tregs in regulating immune and hematological processes.

The present study shows a correlation between the frequency of CD4^+^ T lymphocytes expressing both T-bet and RORγt transcription markers with age and hematological parameters, suggesting a negative effect of age on these cells and their significant negative impact on leucopoiesis and erythropoiesis, contributing to leukemia-associated anemia and recurrent infections. Furthermore, the positive correlation with bone marrow blasts may implicate these cells in the leukemogenesis process, indicating the plasticity of CD4^+^ T cells in AML. While no reports on this specific finding in AML have been identified, T-bet^+^ RORγt^+^ Th17 cells have been observed in autoimmune diseases, as highlighted by [34], where they show plasticity in differentiation and are present in lesional tissue in diseases such as multiple sclerosis and EAE, underscoring the complex nature of Th17 cell lineage specification.

Our study explored the correlation between the frequency (%) and intensity (MFI) of T-bet^+^ CD4^+^ T lymphocytes and RORγt^+^ CD4^+^ T lymphocytes with FAB classification in AML patients. We observed a statistically significant decrease in T-bet^+^ CD4^+^ T lymphocytes’ frequency in M2 AML compared to other FAB groups. This can be due to *RUNX1-RUNX1T1* disrupting MHC class II expression on leukemic blasts and dendritic cells impairing CD4+ T cell activation and TH1 priming [35]. At the same time, M1 AML showed a significant increase in RORγt^+^ CD4^+^ T lymphocyte frequency and RORγt intensity, with a highly significant increase in CD4^+^ T lymphocytes co-expressing T-bet and RORγt. These markers may predict specific FAB classifications, a finding not previously reported in the literature on AML.

In our study, we observed highly significant positive correlations between the expression and intensity (MFI) of the three studied transcription factors (T-bet, RORγt, and FOXP3), indicating a mutually reinforcing effect on each other’s expression. This suggests that these transcription factors may influence each other through autocrine or paracrine mechanisms, direct cell-to-cell interactions, related cytokine activity, or coordinated systemic immune responses. These findings align with the concept presented by Uhl and Gérard [36], who emphasized the importance of coordinated immune responses at a systemic level, where cellular communication plays a crucial role in maintaining pathogen control and self-tolerance. Disruptions in this intercellular communication could potentially lead to immunopathology or autoimmunity, which could also be relevant in the context of AML.

Comparing our findings with [5], who found a positive correlation between Th1 and Th17 cells in newly diagnosed (ND) AML patients, we suggest that similar functional synergism may occur between T-bet and RORγt in AML. This synergy could arise from an immunosuppressive environment, where increased Tregs, dysfunctional NK cells, impaired dendritic cell function, and elevated immunoinhibitory molecules result in immune evasion by the malignant clone. Our data support this hypothesis, as we observed a positive correlation between Treg frequency and relapsed or deceased AML patients, reinforcing the potential role of Tregs in immune suppression within the AML microenvironment.

Additionally, Wang, Hu [24] highlighted the role of TNF-α secreted by Th17 cells in promoting Treg frequency via the TNF-α-TNFR2 pathway in AML. This finding resonates with our results, suggesting that the mutual upregulation of these transcription factors may be part of a feedback loop that facilitates the persistence of immune dysfunction in AML. Overall, our study complements and extends the existing literature on the complex interactions between T-bet, RORγt, and FOXP3 in AML, proposing a possible mechanism by which these transcription factors influence disease progression and immune evasion.

## 5. Conclusions

The observed correlations between T-bet, RORγt, and FOXP3 expression in CD4^+^ T lymphocytes provide critical insights into the immune dysregulation within the bone marrow microenvironment of AML. These findings highlight a potential shift toward inflammation and immunosuppression, contributing to AML pathogenesis and disease progression. Age-related changes in T lymphocyte subsets and the plasticity of CD4^+^ T lymphocytes further emphasize the complexity of immune responses in AML. The significant impact of T-bet^+^ and T-bet^+^ RORγt^+^ CD4^+^ T lymphocytes, as well as RORγt MFI, on bone marrow blast infiltration, thrombopoiesis, and AML-associated anemia, suggests their potential as valuable biomarkers for disease prognosis and therapy. Additionally, the negative correlation of these markers with TLC reflects the underlying immunosuppressive state in AML patients. Increased expression of T-bet and RORγt, along with specific cutoff points for FOXP3 and RORγt frequencies, presents promising predictive markers for AML diagnosis and progression, offering new avenues for clinical monitoring. These results deepen our understanding of AML immunopathogenesis and pave the way for targeted immune-based therapies and early interventions, ultimately benefiting the field by improving patient outcomes and therapeutic strategies.

## Figures and Tables

**Table 1:** Comparison between the control and AML patient groups regarding demographic data.

		Control Group	AML Patients Group	*p*-Value	Sig.
		No. = 25	No. = 80		
Age (years)	Mean ± SD	36.96 ± 3.85	42.89 ± 15.63	0.064	NS
	Range	23–40	18–74		
Gender	Female	8 (32.0%)	30 (37.5%)	0.617	NS
	Male	17 (68.0%)	50 (62.5%)		

**Table 2:** Comparison between the control group and AML patient group regarding hematological laboratory parameters.

		Control Group	Patients Group	*p*-Value	Sig.
		No. = 25	No. = 80		
TLC (×10^3^/mm^3^)	Median (IQR)	6.6 (5.4–7.2)	93 (48.5–142.3)	<0.001	HS
	Range	4.5–8.5	0.04–370		
Hb (g/dL)	Mean ± SD	14.08 ± 0.95	8.45 ± 2.12	<0.001	HS
	Range	12.7–15.4	4.8–14.1		
PLT (×10^3^/mm^3^)	Median (IQR)	231 (198–263)	58 (27–90)	<0.001	HS
	Range	167–345	4–230		
BM BLASTS %	Median (IQR)	1 (0–2)	65 (35–90)	<0.001	HS
	Range	0–5	7–99		

**Table 3:** Comparison between the control group and AML patient group regarding studied markers positive CD4^+^ T lymphocytes % and their MFI.

		Control Group	AML Patients Group	*p*-Value	Sig.
		No. = 25	No. = 80		
T-bet^+^ cells %	Median (IQR)	1.7 (0.4–2.7)	2 (0.8–5.8)	0.088	NS
	Range	0.1–3.4	0.1–10		
T-bet MFI	Mean ± SD	0.95 ± 0.37	2.08 ± 0.83	>**0.001**	**HS**
	Range	0.7–1.9	0.7–5.1		
RORγt^+^ cells %	Median (IQR)	4 (2.1–7)	9.7 (4.2–23)	>**0.001**	**HS**
	Range	1.2–8	0.5–45		
RORγt MFI	Mean ± SD	1.42 ± 0.37	1.26 ± 0.66	0.786	NS
	Range	1.2–2.4	0.3–3		
FOXP3^+^ cells %	Median (IQR)	3.4 (3–5)	59 (38–72)	>**0.001**	**HS**
	Range	1.3–5.3	3.6–87		
FOXP3 MFI	Mean ± SD	2.47 ± 0.71	2.02 ± 1.03	**0.042**	**S**
	Range	1.7–3.4	0.8–5		
Co-expression RORγt and T-bet	Median (IQR)	1.8 (0.4–2)	1 (0.1–5.0)	0.650	NS
	Range	0.1–3	0–12.6		

**Table 4:** Correlation between T-bet^+^ CD4^+^ T lymphocytes percentage and T-bet MFI with other studied parameters in AML patient group.

	T-bet^+^		T-bet MFI	
	r	*p*-Value	r	*p*-Value
Age (years)	−0.298 **	**0.007**	−0.311 **	**0.005**
TLC (×10^3^/mm^3^)	0.017	0.878	0.111	0.329
Hb (g/dL)	−0.244 *	**0.029**	−0.050	0.660
PLT (×10^3^/mm^3^)	0.292 **	**0.008**	−0.180	0.111
BM BLASTS (%)	0.315 **	**0.004**	−0.022	0.849

Spearman correlation coefficients: * significant ** highly significant.

**Table 5:** Correlations between RORγt^+^ CD4^+^ T lymphocytes percentage and RORγt MFI with clinical parameters in AML patients.

	RORγt^+^		RORγt MFI	
	R	*p*-Value	r	*p*-Value
Age (years)	−0.169	0.134	−0.278 *	**0.013**
TLC (×10^3^/mm^3^)	−0.191	0.089	−0.183	0.104
Hb (g/dL)	−0.132	0.244	0.307 **	**0.006**
PLT(×10^3^/mm^3^)	−0.208	0.064	0.024	0.830
BM BLASTS (%)	0.179	0.112	0.349 **	**0.001**

Spearman correlation coefficients: * significant ** highly significant.

**Table 6:** Correlation between FOXP3^+^ CD25^+^ CD4^+^ T lymphocytes % and FOXP3 MFI with the other studied parameters in AML patient group.

	FOXP3^+^		FOXP3 MFI	
	r	*p*-Value	r	*p*-Value
Age (years)	−0.357 **	**0.001**	−0.373 **	**0.001**
TLC (×10^3^/mm^3^)	0.097	0.393	−0.254 *	**0.023**
Hb (g/dL)	−0.184	0.101	−0.111	0.327
PLT (×10^3^/mm^3^)	0.235 *	**0.036**	−0.004	0.974
BM BLASTS (%)	−0.104	0.361	0.059	0.605

Spearman correlation coefficients: * significant ** highly significant.

**Table 7:** Correlation between T-bet^+^ RORγt^+^CD4^+^ T lymphocytes % and studied parameters in the AML group.

Parameter	Co-Expression RORγt & T-bet	
	R	*p*-Value
Age (years)	−0.263 *	0.019
TLC (×10^3^/mm^3^)	−0.296 **	0.008
Hb (g/dL)	−0.268 *	0.016
PLT (×10^3^/mm^3^)	−0.140	0.217
BM BLASTS (%)	0.444 **	<0.001

Spearman correlation coefficients: * significant ** highly significant.

**Table 8:** Relation between T-bet^+^CD4^+^T lymphocytes % and T-bet MFI with the other studied parameters in AML patient group.

		T-bet		*p*-Value	T-bet MFI		*p*-Value
		Median (IQR)	Range		Mean ± SD	Range	
Gender	Female	2.5 (1–5.8)	0.1–6	0.492	2.16 ± 0.67	1–3.4	0.508
	Male	1.9 (0.7–5)	0.1–10		2.03 ± 0.92	0.7–5.1	
FAB	M1	2.4 (2–6) ^a^	1.8–6	**<0.001**	2.21 ± 0.73	1.4–3.4	0.150
	M2	0.2 (0.2–0.2) ^b^	0.1–0.8		1.75 ± 0.46	0.7–2.1	
	M4	2.5 (1–6) ^a^	0.7–10		2.28 ± 1.13	1–5.1	
	M5b	2.35 (1.3–5.8) ^a^	0.1–6		1.88 ± 0.49	1.3–2.8	

Post hoc analysis: Different small superscript letters indicate significant differences between groups.

**Table 9:** Relation between RORγt^+^CD4^+^ T lymphocyte % and RORγt MFI with the other studied parameters in the AML patient group.

		RORγt		*p*-Value	RORγt MFI		*p*-Value
		Median (IQR)	Range		Mean ± SD	Range	
Gender	Female	11.1 (4.3–31)	1–34	0.571	1.31 ± 0.52	0.4–2.2	0.601
	Male	9.7 (4.1–21)	0.5–45		1.23 ± 0.73	0.3–3	
FAB	M1	20 (9.7–33) ^a^	8.2–34	**0.018**	1.74 ± 0.67 ^a^	0.9–3	**0.001**
	M2	12.9 (5–21) ^b^	0.5–23		0.96 ± 0.52 ^b^	0.3–1.8	
	M4	4.3 (4–25) ^b^	1–35.3		1.17 ± 0.69 ^b^	0.4–2.7	
	M5b	7.05 (1.6–9.3) ^b^	1.6–45		1.07 ± 0.35 ^b^	0.4–1.5	

Post hoc analysis: Different small superscript letters indicate significant differences between groups.

**Table 10:** Relation between FOXP3^+^CD25^+^CD4^+^ T lymphocytes% and FOXP3 MFI with the other studied parameters in AML patient group.

		FOXP3^+^ %		*p*-Value	FOXP3 MFI		*p*-Value
		Median (IQR)	Range		Mean ± SD	Range	
Gender	Female	59.5 (38–69)	10.6–87	0.709	2.20 ± 0.85	1.1–4	0.219
	Male	55 (40–72)	3.6–85		1.91 ± 1.12	0.8–5	
FAB	M1	59 (10–77)	5–80	0.519	2.39 ± 1.13	0.8–4	0.071
	M2	54.1 (40–66)	38–66		1.47 ± 0.39	1–1.9	
	M4	60 (44.17–7)	3.6–87		1.94 ± 0.79	1.2–3.9	
	M5b	55 (45–72)	10.6–73		2.12 ± 1.38	1.1–5	

**Table 11:** Relation between T-bet^+^RORγt^+^CD4^+^ T lymphocyte percentage with the other studied parameters in the AML patient group.

		Co-Expression RORγt & T-BET		*p*-Value
		Median (IQR)	Range	
Gender	Female	3.75 (0.1–5.5)	0.1–10	0.182
	Male	0.8 (0.1–2.7)	0–12.6	
FAB	M1	6 (3.5–10) ^a^	1–10	**<0.001**
	M2	0.2 (0.1–0.5) ^b^	0.1–2.7	
	M4	0.5 (0.1–4) ^b^	0–12.6	
	M5b	0.5 (0.1–1) ^b^	0–5.5	

Post hoc analysis: Different small superscript letters indicate significant differences between groups.

**Table 12:** Correlation analysis of FOXP3, RORγt, T-bet expression, and median fluorescence intensity (MFI).

	FOXP3 %		FOXP3 MFI		RORγt %		RORγt MFI		T-bet %		T-bet MFI	
	r	*p*-Value	r	*p*-Value	r	*p*-Value	R	*p*-Value	r	*p*-Value	r	*p*-Value
FOXP3 MFI	0.642 **	**<0.001**										
ROR gamma T %	0.208	0.065	0.533 **	**<0.001**								
ROR gamma T MFI	0.078	0.493	0.420 **	**<0.001**	0.537 **	**<0.001**						
T-bet %	0.258 *	**0.021**	0.340 **	**0.002**	−0.053	0.638	0.148	0.191				
T-bet MFI	0.515 **	**<0.001**	0.503 **	**<0.001**	0.411 **	**<0.001**	0.343 **	**0.002**	0.238 *	**0.034**		
Co-expression RORγt & T-bet %	0.399 **	**<0.001**	0.586 **	**<0.001**	0.565 **	**<0.001**	0.411 **	**<0.001**	0.537 **	**<0.001**	0.308 **	**0.005**

Spearman correlation coefficients: * significant ** highly significant.

## Data Availability

All data generated in this study are presented in the current MS.
